# Efficacy of herbal medicine treatment based on syndrome differentiation for Parkinson’s disease: A systematic review and meta-analysis of randomized placebo-controlled clinical trials

**DOI:** 10.3389/fphar.2023.1108407

**Published:** 2023-02-27

**Authors:** Purumea Jun, HuiYan Zhao, In Chul Jung, Ojin Kwon, Chang-Hyun Han, Jiyoon Won, Jung-Hee Jang

**Affiliations:** ^1^ KM Science Research Division, Korea Institute of Oriental Medicine, Daejeon, Republic of Korea; ^2^ University of Science & Technology, Campus of Korea Institute of Oriental Medicine, Korean Convergence Medical Science Major, Daejeon, Republic of Korea; ^3^ Department of Oriental Neuropsychiatry, College of Korean Medicine, Daejeon University, Daejeon, Republic of Korea; ^4^ Department of Meridian & Acupoint, College of Korean Medicine, Dong-Eui University, Busan, Republic of Korea

**Keywords:** Parkinson’s disease, syndrome differentiation, herbal medicine, systematic review, meta-analysis

## Abstract

**Background:** Parkinson’s disease (PD), the second most common progressive neurodegenerative disease, causes heterogeneous clinical symptoms. Patients experience a range of motor and non-motor symptoms, and personalized diagnosis and treatment are needed. In traditional East Asian medicine, syndrome differentiation (SD) is a diagnostic approach for customized therapy that uses a comprehensive analysis and varies for the same disease. We aimed to evaluate the efficacy of herbal medicine (HM) prescribed according to the SD of PD.

**Methods:** Ten electronic databases were searched from inception to August 2021 without language limitations. All randomized controlled trials (RCTs) involving HM for SD of PD were included. Assessment of Cochrane's risk of bias and meta-analysis and Grading of Recommendations Assessment, Development, and Evaluation was also performed. Effect measurement was summarized using the mean difference (MD) with 95% confidence interval, through a meta-analysis.

**Results:** Thirteen RCTs involving 843 participants were included. The overall risk of bias was either low or unclear. Compared with the placebo, a combined therapy of HM and Western medicine (WM) significantly improved the total Unified Parkinson’s Disease Rating Scale (UPDRS) (MD = −8.03, [−10.27, −5.79], *p* < 0.00001; I^2^ = 0%) and was more beneficial, as assessed using the UPDRS (I–III), the Parkinson’s Disease Questionnaire-39, and the Non-Motor Symptoms Scale. Adverse events did not differ between the groups.

**Conclusion:** The findings suggest that the combined treatment of WM and HM based on SD diagnosis has additional benefits in PD treatment. However, the methodological quality of the included RCTs was suboptimal. Nevertheless, this systematic review is the first to investigate the efficacy of HM treatment according to the SD diagnosis in PD. The clinically meaningful improvement in HM according to SD in PD needs to be tested in further studies with rigorous designs and longer follow-up periods.

**Systematic Review Registration**: [https://inplasy.com/inplasy-2021-10-0020/], identifier [INPLASY2021100020].

## 1 Introduction

Parkinson’s disease (PD) is the second most common neurodegenerative disease, caused by progressive degeneration of neurons or their myelin sheaths. It is mainly characterized by deposition of proteins with altered physicochemical properties in the brain and peripheral organs (Postuma et al., 2015). PD is characterized by various physical signs, including motor and non-motor manifestations, which involve a multitude of functions (Alberio et al., 2010). Current conventional treatments are based on dopamine replacement therapy and reduction of dopamine degradation. Although dopaminergic medications are the standard treatment for drastic improvement in motor symptoms and quality of life in patients with PD, the treatment effects become increasingly less beneficial and progressively more disabled due to the wearing-off of levodopa effects caused by long-term usage (Poewe et al., 2017). Furthermore, L-DOPA-related motor complications include motor fluctuations and L-DOPA-induced dyskinesia, adventitious involuntary movements, and L-DOPA-resistant motor features, including treatment-resistant tremor, freezing of gait, postural instability and falls, and swallowing and speech disturbances (Tambasco et al., 2018). Some non-motor symptoms do not respond to dopamine replacement therapies as much as motor symptoms (Lee and Koh, 2015). Therefore, the demand for complementary and alternative medicine for conventional therapy in PD is increasing.

Traditional East Asian medicine (TEAM) has been reportedly based on holism to treat PD, with few side effects (Kim et al., 2012; Liu et al., 2020). Treatment based on syndrome differentiation (SD) is the basic principle of illness and treatment in TEAM, which uses four diagnostic criteria, namely, observation, listening, questioning, and checking the pulse condition (Jiang et al., 2012). It can improve symptoms and provide personalized treatment plans that are scientific and superior (Jiang et al., 2012). Especially, SD diagnosis is classified as variable TM syndrome in the same disease and guides individualized clinical medication (Wang and Xu, 2014). Therefore, individual patients with PD show several clinical symptoms and disease progressive courses, and herbal medicine (HM) treatment according to the SD diagnosis will benefit PD. Many previous studies have reported that HM can effectively improve motor and non-motor symptoms in PD, and evidence suggests the potential superiority of complementary use of HM for PD treatment (Chung et al., 2006; Kim et al., 2012; Zhang et al., 2014; Shan et al., 2018; Liu et al., 2020). However, there are no systematic reviews (SRs) that assessed the effectiveness of herbal medicine treatment according to SD in PD. Therefore, investigation of the efficacy and safety of herbal medicine based on SD as a personalized treatment for PD is needed. This approach is increasingly required to overcome the limitations of dopaminergic medication.

Previous reviews (Wang et al., 2012; Zhang et al., 2015) confirmed the insufficiency of evidences that support the use of HM for patients with PD. Moreover, generally low-quality studies were included. Recently, a study (Shan et al., 2018) that exclusively included randomized double-blind placebo-controlled clinical trials has also been published; however, SD has not been considered. This study aimed to compare the effects of HM on PD based on SD. We adopted a conventional SR and meta-analysis of randomized placebo-controlled clinical trials that included high-quality studies and compared the effect sizes of various HMs to help make decisions regarding the management of patients with PD. We also checked the symptom changes in the HM of SD.

## 2 Materials and methods

### 2.1 Study registration

During all phases of the study design and implementation of this SR, we adhered to the preferred reporting items for systematic reviews and meta-analyses statement guidelines and those of our previous study. A checklist is presented in Supplementary Table S1. The protocol has been published in Evidence-Based Complementary and Alternative Medicine (Jun et al., 2022). It has been registered in the International Platform of Registered Systematic Review and Meta-Analysis Protocols (INPLASY) 2021 under the registration number INPLASY2021100020.

### 2.2 Database and Search Strategy

Databases and search terms were determined through discussions among three authors (PJ, H-YZ, and J-HJ) before the literature search was executed. The following electronic databases were searched from their inception to August 2021. The electronic databases included three core databases (PubMed, Cochrane Central Register of Controlled Trials (CENTRAL), and Embase); four Chinese databases (China National Knowledge Infrastructure, Wanfang database, China Science and Technology Journal Database, and China Biology Medicine disc); and three Korean databases (Korea Citation Index, Korean Studies Information Service System, and Oriental Medicine Advanced Searching Integrated System). The following keywords were used as search terms: PD (e.g., “Parkinson’s disease [Mesh terms]” or “Parkinson” [Title/Abstract]"); SD (e.g., “pattern [Title/Abstract]" or “syndrome [Title/Abstract]"); and HM (herbal medicine” [Title/Abstract] or “Korea medicine” [Title/Abstract]). The all-search strategy is described in the supplementary file of our previous protocol (Jun et al., 2022).

### 2.3 Study selection

#### 2.3.1 Type of studies

Only parallel randomized controlled trials (RCTs) on HM in PD with published studies were eligible for inclusion. Trials were excluded based on the following criteria: animal studies, case reports/series, literature review, and non-parallel RCTs.

#### 2.3.2 Type of participants.

Participants of any age and sex with idiopathic PD were included in this study. Diagnosis of PD should be performed considering clinical symptoms and radiological examinations using standard diagnostic criteria, such as the Chinese National Diagnostic Standard in 2006 for PD (Liu, 2016) or UK PDS Brain Bank criteria (Hughes et al., 1992). Moreover, only patients diagnosed with SD were included in the study.

#### 2.3.3 Types of intervention

HM was combined with conventional therapies such as Western medicine (WM). HM was administered regardless of the formula, the form of administration, dosage, frequency, or treatment duration. WM included Madopar and Sinemet for conventional PD or amantadine and piribedil.

#### 2.3.4 Types of comparisons

To improve the quality of this review, the comparison intervention only included a placebo combination with WM.

#### 2.3.5 Treatment method

There is no restriction on the dosage, including the frequency, dose, intensity, and duration.

#### 2.3.6 Outcome measure

The primary outcome was the Total Unified Parkinson’s Disease Rating Scale (UPDRS). The UPDRS I (mental dysfunction and mood), UPDRS Ⅱ (activities of daily living), UPDRS Ⅲ (motor section), and UPDRS IV (assessment of treatment-related motor and non-motor complications) (Movement Disorder Society Task Force on Rating Scales for Parkinson’s Disease, 2003). Parkinson’s Disease Questionnaire-39 (PDQ-39) (assessment of the impact of the treatment area upon particular aspects of the function and well-being in patients with PD) (Hagell and Nygren, 2007); the Non-Motor Symptoms Scale (NMSS) (comprehensive assessment of a range of non-motor symptoms in PD) (Chaudhuri et al., 2005); clinical symptoms (limb spasm, stiff neck, insomnia, night sweats, and back and leg pain); and adverse events (AEs) were the secondary outcomes.

### 2.4 Data extraction

Three authors independently extracted the data. For each study, the following variables were extracted: author information, year of publication, treatment regimen (HM and HM ingredient), control intervention, sample size, inclusion and exclusion criteria, information regarding SD, treatment period, side effects, and primary and secondary outcome measurements. All Korean or Chinese to English translations were deduced primarily from the World Health Organization (WHO)’s international standard terminologies (WHO, 2007). Any discrepancies in cross-checking were resolved through discussion.

### 2.5 Assessment for risk of bias in included studies

The risk of bias used was from the Cochrane Collaboration’s tool for the SR of intervention (Higgins, 2011). Two investigators conducted all assessment processes (PJ and H-YZ), independently. This tool consists of seven domains: selection bias (random sequence generation and allocation concealment); performance bias (blinding of participants and personnel); detection bias (blinding of outcome assessment); attrition bias (incomplete outcome data); reporting bias (selective outcome reporting); and other biases. A third reviewer resolved the disagreement. The details of the assessment measure are shown in Supplementary Table S2.

### 2.6 Statistical analysis

#### 2.6.1 Measures of the treatment effect

The Review Manager (version 5.4) software of the Cochrane Collaboration was used for data analysis (RevMan, the Cochrane Collaboration, London, England, 2020). Data for continuous outcomes were summarized using the mean difference (MD). Meanwhile, dichotomous outcomes are expressed as risk ratios (RRs) with 95% confidence intervals. Statistical heterogeneity was analyzed using I^2^ and χ^2^ tests. I^2^ was 0%–50%, which means low; 50%–75%, meaning serious; and >75%, meaning very serious. For the analysis model, if *p* > 0.1 and I^2^ < 50%, the meta-analysis will use the fixed-effect model; otherwise, the random-effect model was used. A subgroup analysis was conducted to assess the specific effectiveness of the same SD, which was used in the identical original SD naming in the articles.

#### 2.6.2 Frequency analysis

The frequencies of SD, HM, and HM ingredients were calculated to investigate the SD distribution and assess the herbal ingredient.

#### 2.6.3 Dealing with missing data

If there were missing data, the authors attempted to obtain the necessary information by contacting the first or corresponding authors of the included trials by phone, email, or fax.

#### 2.6.4 Assessment of the quality of evidence

The Grading of Recommendations Assessment, Development, and Evaluation (GRADE) method assessed the quality of evidence for primary and secondary outcomes (Schünemann et al., 2016). The overall GRADE assessment was divided into high, moderate, low, and very low scores. The details of the GRADE assessment are shown in Supplementary Table S3

## 3 Results

### 3.1 Study selection and characteristics

**FIGURE 1:**
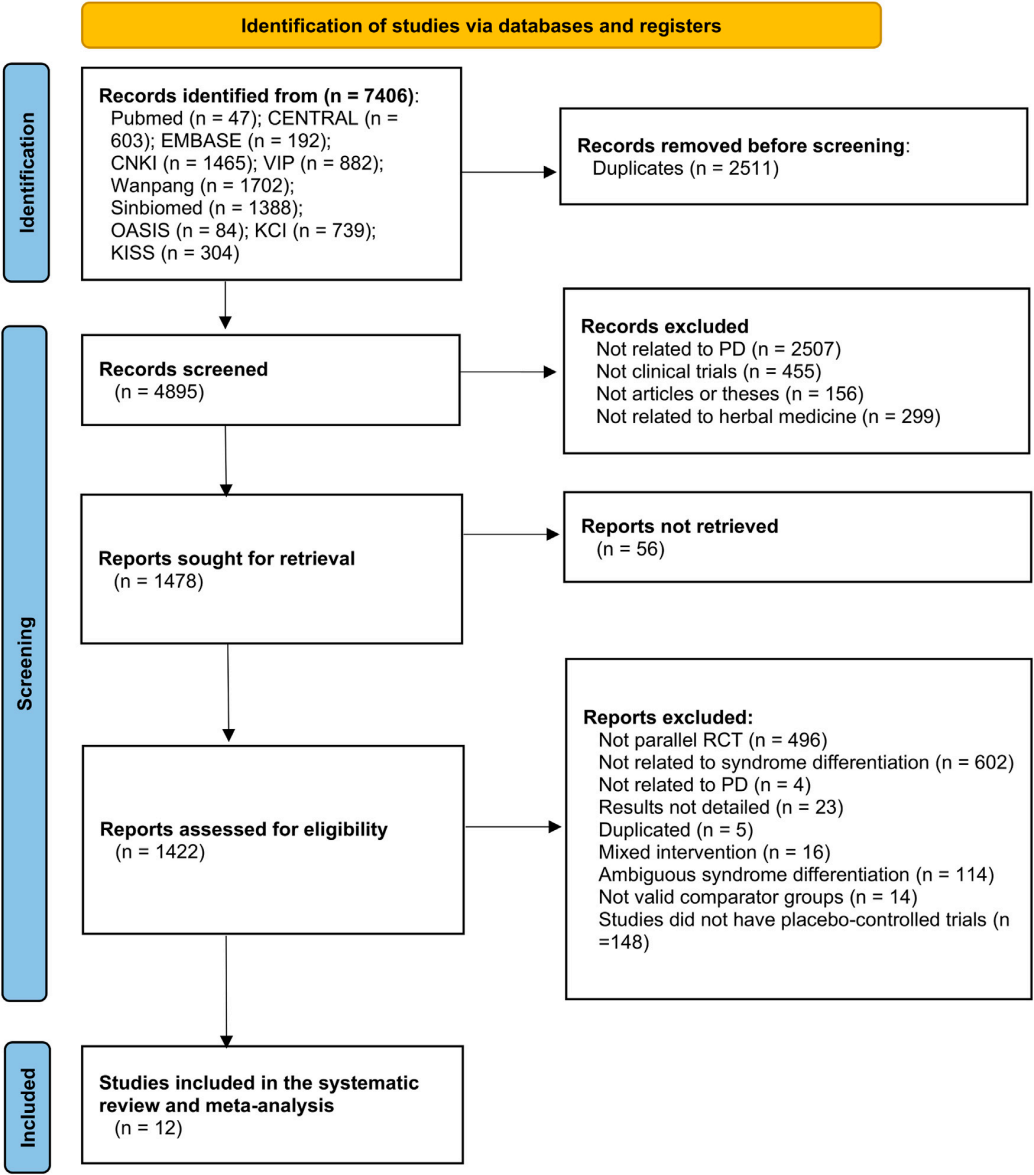
PRISMA flow diagram of the study selection process. PD, Parkinson’s disease; RCT, randomized controlled trial.

The data searches yielded 47 studies from PubMed, 603 from CENTRAL, 192 from Embase, 1,465 from CNKI, 882 from VIP, 1702 from Wanfang, 1,388 from Sinbiomed, 84 from OASIS, 739 from KCI, and 304 from KISS. After the duplicates were removed, 4,895 studies remained. Based on the title and abstract screening, 1,478 studies remained. A total of 1,478 studies were selected for full-text review and data processing, and 1,422 studies were excluded, including 496 that were not parallel RCTs, 602 that were not SD, four that were related to PD, 23 that did not have detailed results, five that were duplicates, 114 that were ambiguous SD, 16 that had mixed interventions, 14 that included valid comparator groups, and 148 that were not placebo-controlled trials. Ultimately, 12 RCTs were included in this meta-analysis (Figure 1).

### 3.2 Study characteristics

The characteristics of the included studies are presented in Table 1. All trials were conducted in China. Eleven of them were written in Chinese (Jin, 2004a; Jin, 2004b; Ma, 2008; Xiao, 2010; Chen et al., 2014; Zeng, 2015; Yu, 2016; Yang, 2017; Liu, 2018; Ma, 2018; Lin et al., 2019), and one was written in English (Chen et al., 2020). A total of 843 patients with PD were included in this meta-analysis, with sample sizes ranging from 24 to 108 (median 34.5). The disease duration ranged from 2.7 to 7.1 years (median 4.8). The duration of treatment ranged from 3 weeks to 3 months (median, 12 weeks). Two studies focused on non-motor symptoms, including non-motor and sleep disorders (Liu, 2018; Lin et al., 2019).

**TABLE 1:** Characteristics of included studies.

First author	Syndrome differentiation	Major target	Intervention group (n)	Control group (n)	Course of treatment	Outcome index	Intergroup differences
Country (year)		H and Y stage					
		Mean age					
		Course of disease (intervention/control years)					
Chen et al. (2014)	Liver–kidney deficiency and dual deficiency of Qi and blood	PD 1–3 66.4/65.6 4.4/4.4	Zhi Chan (one package, bid) plus Madopar or Sinemet (NR); (n = 57)	Placebo plus Madopar or Sinemet (NR); (n = 51)	3 months	1. Total UPDRS; 2. UPDRS Ⅰ; 3. UPDRS Ⅱ; 4. UPDRS Ⅲ; 5. UPDRS Ⅳ; 6. TCM symptom score	1. *p* < 0.05; 2. NR; 3. NR; 4. *p* < 0.05; 5. NR 6. *p* < 0.05
Chen et al. (2020)	Kidney essence deficiency	PD 1–4 64.1/65.2 5.1/4.8	Cong Rong Shu Jing (0.5 package, bid) plus WM (NR); (n = 35)	Placebo plus WM (NR); (n = 37)	3 months	1. Total UPDRS; 2. UPDRS Ⅰ; 3. UPDRS Ⅱ; 4. UPDRS Ⅲ; 5. UPDRS Ⅳ; 6. TCM syndrome score; 7. levodopa equivalent dose	1. *p >* 0.05; 2. *p* < 0.05; 3. *p* < 0.05; 4. *p* < 0.05; 5. *p* < 0.05
Jin, (2004a)	Liver–kidney yin deficiency and stirring of wind	PD 1–4 65.3/61.6 5.3/5.0	Nao Kang Ning (three capsules, tid) plus conventional PD WM (NR); (n = 30)	Placebo plus conventional PD plus WM (NR); (n = 30)	3 months	1. UPDRS Ⅲ; 2. TCM symptom score; 3. SF-36; 4. dosage of WM	1. *p* < 0.05; 2. *p* < 0.05; 3. *p* < 0.05; 4. *p* < 0.05
Jin, (2004b)	Liver–kidney yin deficiency and stirring of wind	PD 1–4 66.8/64.2 4.2/3.7	Pa An (three capsules, tid) plus conventional PD of WM (NR); (n = 35)	Placebo plus conventional PD WM (NR); (n = 35)	3 months	1. Total UPDRS; 2. TCM symptom score; 3. SF-36; 4. dosage of WM	1. *p >* 0.05; 2. *p* < 0.05; 3. *p* < 0.05; 4. *p* < 0.05
Lin et al. (2019)	Phlegm-heat stirring wind, and blood stasis with wind-stirring	PD with NMS 1–4 56.3/54.8 7.1/6.8	Tian Dan Tong Luo (2 g, tid) plus Madopar (0.25 g); (n = 53)	Placebo plus Madopar (0.25 g); (n = 51)	3 weeks	1. TCM syndrome score; 2. total UPDRS score; 3. PDQ-39; 4. NMSS	1. *p* < 0.05; 2. *p* < 0.05; 3. *p* < 0.05; 4. *p >* 0.05
Liu, (2018)	Liver–kidney yin deficiency	PD with NMS (sleep disorder) 1.5–4 67.7/67.6 67.2/57.6 (month)	Bu Shen Zhi Chan An Shen (0.5 doses, bid) plus conventional PD of WM (NR); (n = 29)	Placebo plus conventional PD WM (NR); (n = 29)	1 month	1. UPDRS Ⅲ; 2. PSQI; 3. ESS; 4. PDSS; 5. clinical efficacy	1. *p <* 0.05; 2. *p* < 0.05; 3. *p* < 0.05; 4. *p* < 0.05; 5. *p* < 0.05
Ma, (2008)	Liver–kidney yin deficiency	PD NR 66.5/65.8 5.9/5.8	Xi Feng Ding Chan (6 g, tid) plus Madopar (NR); (n = 40)	Placebo plus Madopar (NR); (n = 40)	3 months	1. Clinical efficacy; 2. total UPDRS; 3. SF-36; 4. drug adverse reaction	1. *p* < 0.01; 2. *p* < 0.05; 3. *p* < 0.05; 4. *p* < 0.01
Ma, (2018)	Liver–kidney yin deficiency	PD 1–3 67.7/68.7 3.9/4.5	HM (0.5 doses, bid) plus conventional PD plus WM (NR); (n = 30)	Placebo plus conventional PD plus WM (NR); (n = 30)	2 months	1. Total UPDRS; 2. UPDRS Ⅰ; 3. UPDRS Ⅱ; 4. UPDRS Ⅲ; 5. TCM symptom score; 6. CCS	1. *p <* 0.05; 2. *p* < 0.05; 3. *p* < 0.05; 4. *p >* 0.05; 5. *p <* 0.01; 6. *p <* 0.01
Xiao, (2010)	Blood stasis with wind-stirring or liver–kidney yin deficiency	PD 1–4 64.3/63.4 5.0/4.0	Bu Shen Huo Xue (bid) plus Madopar (375–1,000 mg/d 3–4 t/d), amantadine (100–200 mg/d 1–3 t/d), and piribedil (0.5–2 pills, 1–2 t/d); (n = 12)	Placebo plus Madopar (375–1,000 mg/d 3–4 t/d), amantadine (100–200 mg/d 1–3 t/d), and piribedil (0.5–2 pills, 1–2 t/d); (n = 12)	3 months	1. Total UPDRS; 2. UPDRS Ⅱ; 3. UPDRS Ⅲ; 4. UPDRS Ⅳ; 5. PDSS; 6. PDQ-39	1. *p* > 0.05; 2. *p >* 0.05; 3. *p >* 0.05; 4. *p >* 0.05; 5. *p >* 0.05; 6. *p >* 0.05
Yang, (2017)	Liver–kidney deficiency, wind-phlegm, and blood stasis blocking	PD NR 65.2/63.3 7.0/6.2	Yi Shen Chu Chan (0.5 doses, bid) plus Madopar (NR); (n = 40)	Placebo plus Madopar (NR); (n = 39)	2 months	1. UPDRS Ⅲ; 2. PDQ-39; 3. NMSS; 4. MDRSPD; 5. TCM symptom scale	1. *p* < 0.05; 2. *p* < 0.01; 3. *p* < 0.01; 4. *p >* 0.05; 5. *p* < 0.05
Yu, (2016)	Blood stasis with wind-stirring	PD 1–3 70.8/69.7 24.2/26.0 (months)	Nao Kang (one package, tid) plus Madopar (125 mg, tid); (n = 34)	Placebo plus Madopar (125 mg, tid); (n = 34)	2 months	1. UPDRS Ⅱ; 2. UPDRS Ⅲ; 3. NMSS; 4. TCM syndrome score; 5. H and Y stage	1. *p* < 0.05; 2. *p* < 0.05; 3. *p* < 0.05; 4. *p* < 0.05; 5. *p* < 0.05
Zeng, (2015)	Internal stirring of endogenous wind, phlegm, and blood stasis block collaterals	PD 1–3 67.7/66.9 2.7/2.7	Zhen Chan (one package, bid) plus Madopar (500–1,000 mg) and conventional PD WM (NR); (n = 30)	Placebo plus Madopar (500–1,000 mg) and conventional PD WM; (n = 30)	2 months	1. Total UPDRS; 2. clinical symptoms of TCM; 3. dose of WM	1. *p* < 0.05; 2. *p* < 0.05; 3. *p* < 0.05

CCS, Cleveland Constipation Score; ESS, Epworth Sleepiness Scale; H and Y grade, Hoehn and Yahr grade; MDRSPD, Parkinson’s Disease Motor Function Scale; NMS, Non-Motor Symptom; NR, no report; PD, Parkinson’s disease; PDQ-39, Parkinson’s Disease Questionnaire-39; PQSI, Pittsburgh Sleep Quality Index; TCM, traditional Chinese medicine; UPDRS, Unified Parkinson’s Disease Rating Scale; WM, Western medicine.

**FIGURE 2:**
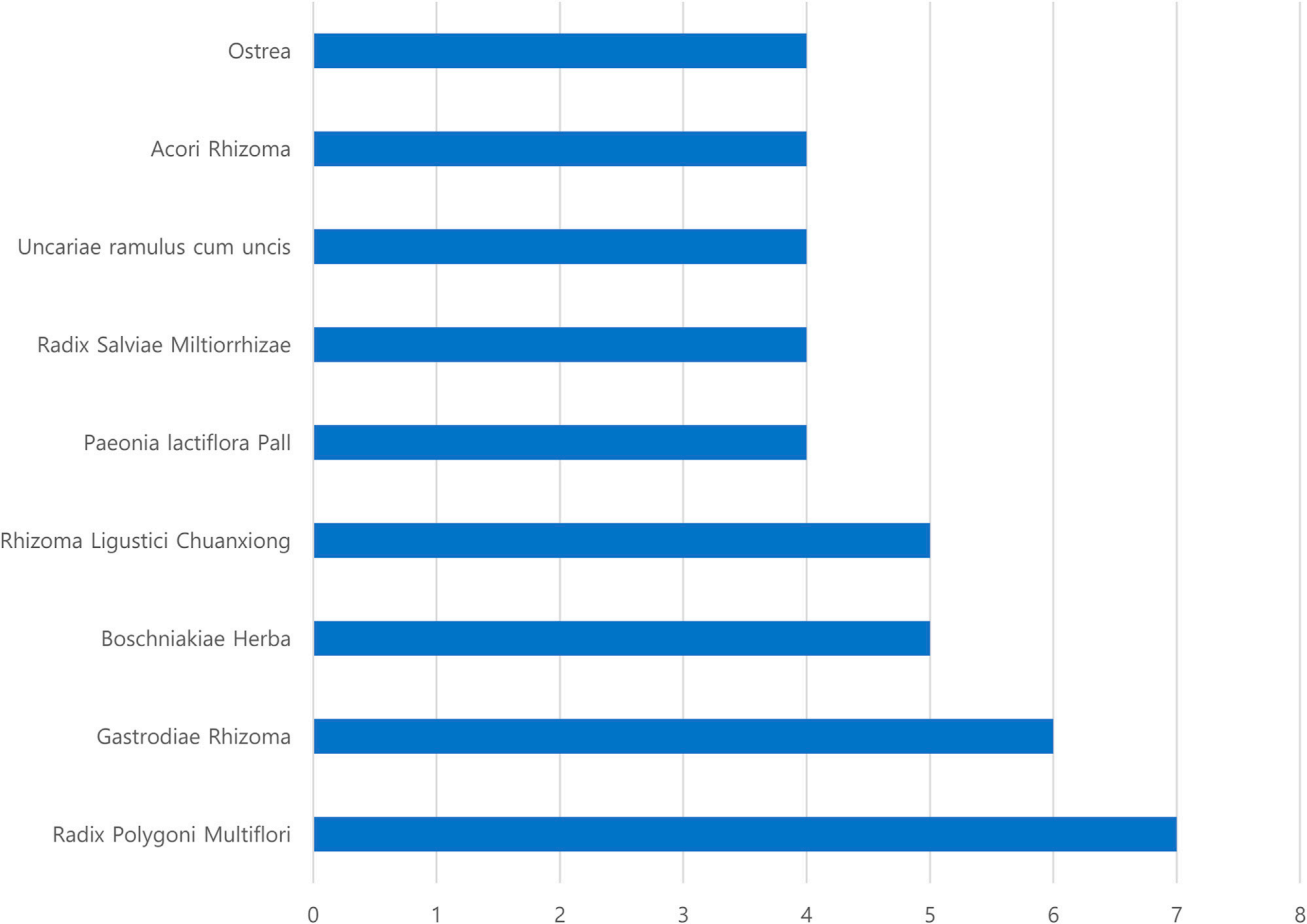
Frequency of commonly used herbs in herbal formulas for treating patients with PD.

Frequency analysis of the herbs used in the herbal formulas for treating patients with PD according to SD was conducted (Table 2). The results showed 10 SD and 13 herbal formulas (one formula used the same herbal ingredients with different names). The most frequent SD was “liver–kidney yin deficiency,” used in three studies (Ma, 2008; Liu, 2018; Ma, 2018). In addition, the frequency of herbal ingredients was computed. The top four herbal ingredients were *Radix Polygoni Multiflori*, *Gastrodis Tuber*, *Boschniakiae Herba*, *and Rhizoma Ligustici Chuanxiong* (Figure 2).

**TABLE 2:** Herbal medicine and pattern identification for treating PD.

Syndrome differentiation	Name of herbal formula	Composition of herbal formula (Latin name)
Liver–kidney deficiency and dual deficiency of Qi and blood	Zhi Chan Granules	*Astragalus membranaceus, Uncariae Ramulus Cum Uncis, Radix Polygoni Multiflori, Praeparata, Paeonia lactiflora Pall,* and *Anemarrhenae asphodeloides rhizoma*
Kidney essence deficiency	Cong Rong Shu Jing Granules	*Boschniakiae Herba, Cortex Moutan,* and *Salvia miltiorrhiza Bge*
Liver–kidney yin deficiency and stirring of wind	Nao Kang Ning Capsule	*Radix Polygoni Multiflori 0.4 g, Gastrodiae rhizome 0.4 g, Uncariae Ramulus Cum Uncis 0.4 g, Radix Rehmanniae Recens 0.4 g,* and *Ligusticum striatum 0.4 g*
	Pa’an Capsule	*Radix Polygoni Multiflori 0.4 g, Gastrodiae rhizome 0.4 g, Uncariae Ramulus Cum Uncis 0.4 g, Radix Rehmanniae Recens 0.4 g,* and *Ligusticum striatum 0.4 g*
Phlegm-heat stirring wind and blood stasis with wind-stirring	Tian Dan Tong Luo Capsule	*Radix Salviae miltiorrhizae, Rhizoma Ligustici Chuanxiong, Bdelloidea, Herba Siegesbeckiae, Achyranthis Bidentatae Radix, Styphnolobium japonicum, Bos taurus domesticus Gmelin, Acorus tatarinowii, Astragali Radix, Orientalis,* and *Gastrodiae Rhizoma*
Liver–kidney yin deficiency	Bu Shen Zhi Chan An Shen Fang	*Cornu Cervi Degelatinatum 10 g, Radix Rehmanniae Recens 25 g, Cannabis Fructus 20 g, Codonopsis pilosula 20 g, Boschniakiae Herba 30 g, Asini Corii Colla 10 g, Cornus officinalis Sieb 10 g, Ophiopogonis Radix 15 g, Lycii Fructus 30 g, Gastrodiae rhizome 12 g, Paeonia lactiflora Pall 30 g, Uncariae Ramulus Cum Uncis 30 g, Carapax Pelochelydis 15 g, Carapax Testudinis 20 g, Concha Ostreae 30 g, Mastodi Fossilia Ossis 30 g, Radix Polygoni Multiflori 30 g, Albiziae Cortex 15 g, Ziziphi spinosae semen 15 g,* and *Glycyrrhizae Radix et Rhizoma Praeparata 6 g*
	Xi Feng Ding Chan Wan	*Carapax Testudinis, Radix Polygoni Multiflori, Praeparata, Gastrodiae rhizome, Bombyx batryticatus, Paeonia lactiflora Pall, Ligusticum striatum,* and *Acorus tatarinowii*
	No specific name	*Radix Polygoni Multiflori 15 g, Achyranthes bidentata Blume 15 g, Cornus officinalis Sieb 12 g, Dioscorea opposita Thunb 12 g, Carapax Testudinis 12 g, Angelica sinensis 12 g, Scrophularia ningpoensis Hemsl 12 g, Rehmannia glutinosa Libosch 12 g, Cistanche salsa 15 g, Cannabis sativa L 10 g, Platycladus orientalis 10 g,* and *Glycyrrhiza uralensis Fisch 6 g*
Blood stasis with wind-stirring or liver–kidney yin deficiency	Bu Shen Huo Xue Ke Li	*Fructus Corni, Radix Polygoni Multiflori, Angelica sinensis, Lycium barbarum L, Epimedii Folium, Boschniakiae Herba, Acorus tatarinowii, Scolopendra dehaani,* and *Salviae Radix*
Liver–kidney deficiency, wind-phlegm, and blood stasis blocking	Yi Shen Chu Chan Tang	*Polgoni Multiflori Radix Praeparata 30 g, Rehmannia glutinosa Libosch 20 g, Gastrodiae Rhizoma 20 g, Cynanchum otophyllum Schneid 20 g, Ostreae Concha 30 g, Bombyx Batryticatus 10 g, Rheum officinale Baill 6 g, Lindera aggregata 20 g, Rhizoma Dioscoreae 20 g, Alpinia oxyphylla* and *Miq 20 g*
Blood stasis with wind-stirring	Nao Kang Ke Li	*Boschniakiae Herba, Panax notoginseng, Cnidii Rhizoma, Salviae Radix, Acorus tatarinowii, Polygala tenuifolia Willd, Scolopendra dehaani, Pheretima, Bombyx Batryticatus, Scorpio, Astragali Radix, Codonopsis pilosula,* and *Epimedium Linn*
Internal stirring of endogenous wind, phlegm, and blood stasis block collaterals	Zhen Chan Ke Li	*Cynanchum otophyllum Schneid 30 g, Boschniakiae Herba 20 g, Rhodiola rosea L 20 g, Pulsatilla chinensis 20 g, Gleditsia Simensislam 3 g, Bombyx Batryticatus 10 g, Cryptotympana pustulata Fabr 5 g, Curcuma longa L 15 g, Rheum palmatum 5 g, Polygalae Radix,* and *Preparata Cum Glycyrrhizae Radix 5 g*

### 3.3 Quality of the included studies

**FIGURE 3:**
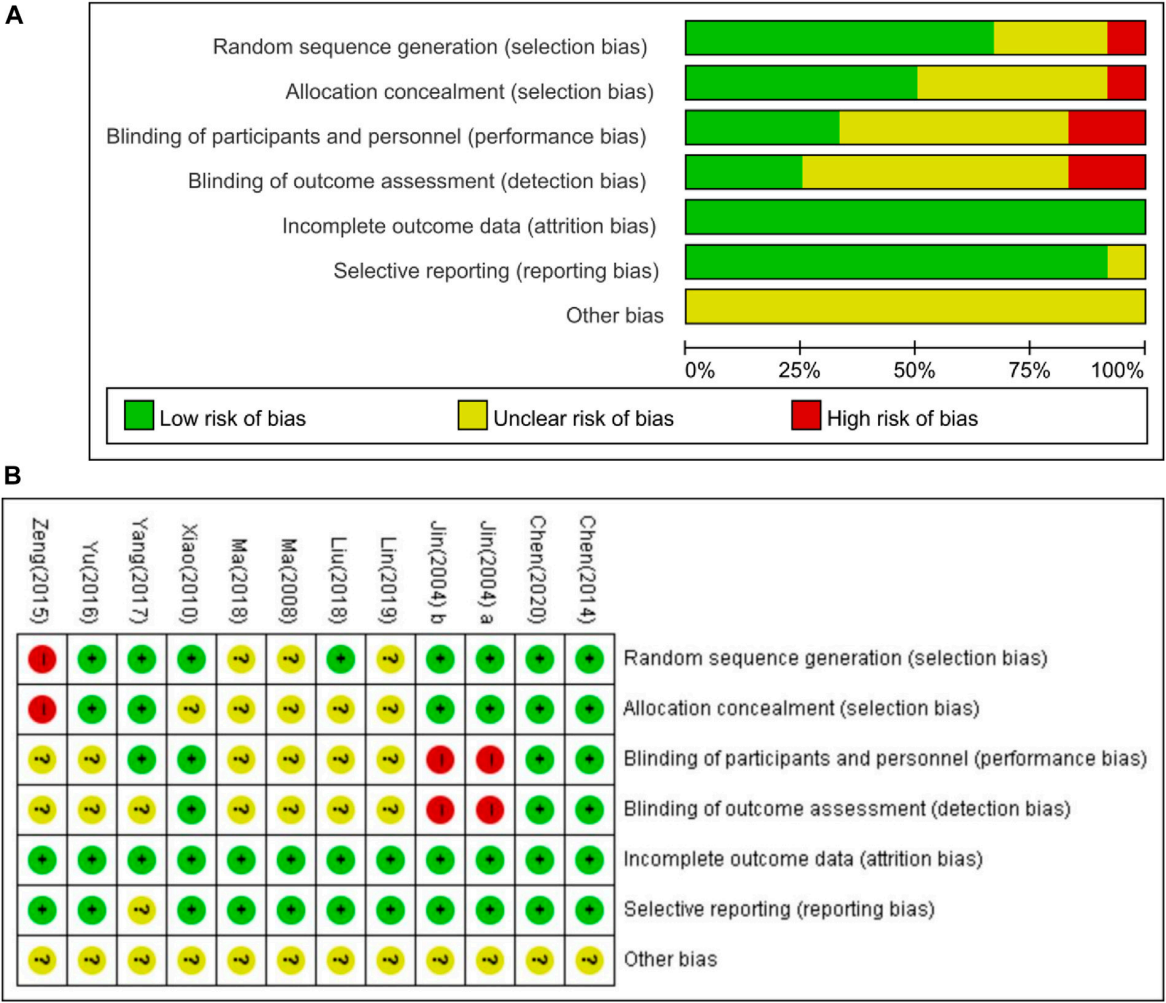
Risk of bias for included studies. **(A)** Risk of bias summary; **(B)** risk of bias graph. Green, low risk of bias; red, high risk of bias; yellow, unclear risk of bias.

Figure 3 shows the risk of bias in the included studies. All studies mentioned randomization in the trials. However, one study used a visiting sequence, which was assessed as a high risk of bias for random sequence generation (Zeng, 2015). Three studies did not mention a specific generation method, resulting in an unclear risk of bias (Ma, 2008; Ma, 2018; Lin et al., 2019). Eight studies used computer programs to list random numbers (Jin, 2004a; Jin, 2004b; Xiao, 2010; Chen et al., 2014; Yu, 2016; Yang, 2017; Liu, 2018; Chen et al., 2020). Six studies reported allocation concealment (Jin, 2004a; Jin, 2004b; Chen et al., 2014; Yu, 2016; Yang, 2017; Chen et al., 2020). Two studies used single binding, which is considered to have a high risk of bias for blinding participants and personnel (Jin, 2004a; Jin, 2004b). All studies were assessed as having a low risk of bias for incomplete outcome data. One study had an unclear risk of bias in selection reporting because of the study protocol (Yang, 2017). None of them stated that the sample size calculation led to an unclear risk of bias.

### 3.4 Synthesis of results for the effectiveness and safety of HM

#### 3.4.1 Effectiveness

**FIGURE 4:**
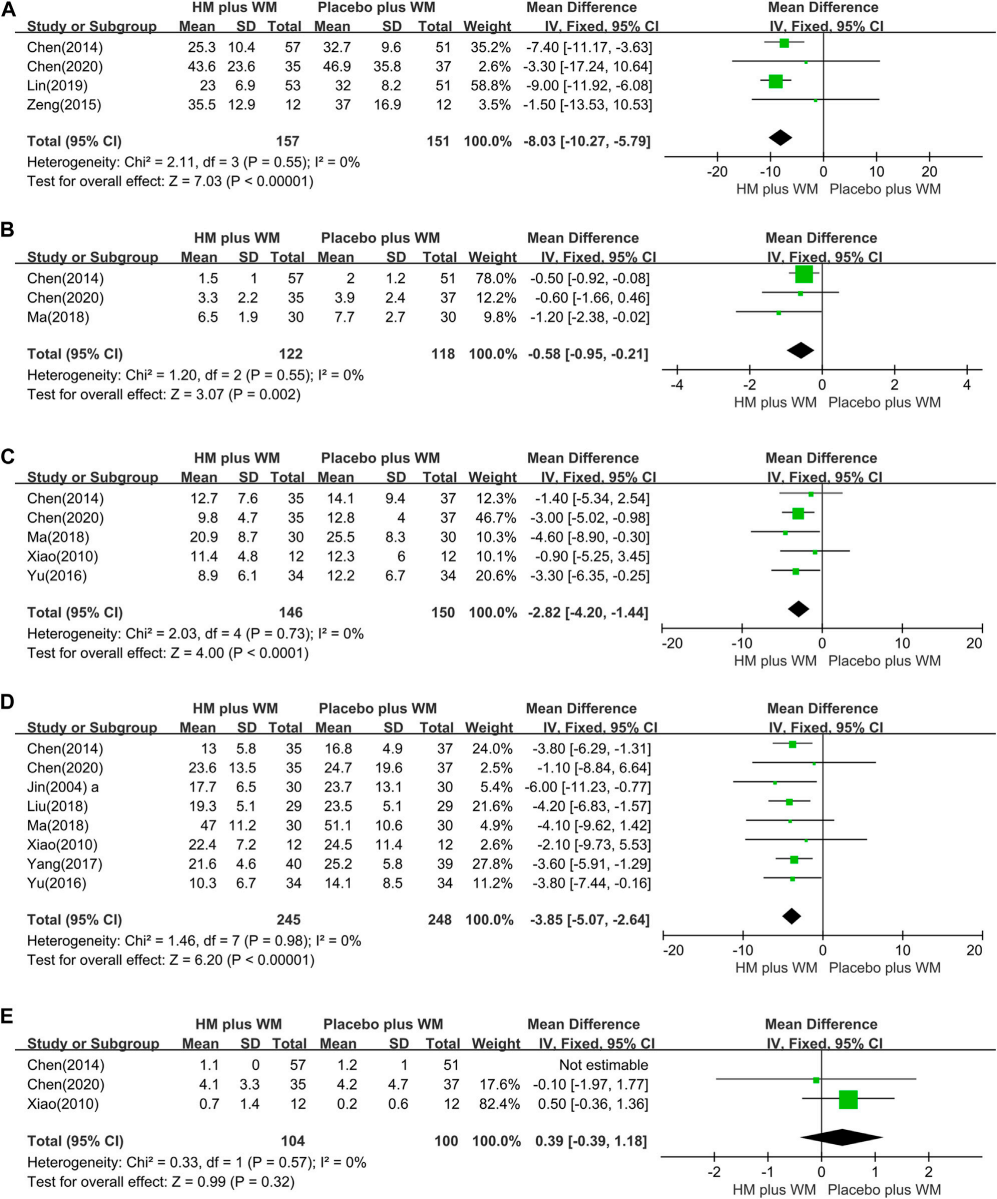
Forest plot of HM plus WM versus placebo plus WM. **(A)** Total UPDRS, **(B)** UPDRS Ⅰ, **(C)** UPDRS Ⅱ, **(D)** UPDRS Ⅲ, and **(E)** UPDRS Ⅳ.

*Total UPDRS*: Four studies (Chen et al., 2014; Zeng, 2015; Lin et al., 2019; Chen et al., 2020) including 508 participants (157 in the experimental group and 151 in the comparison group) were included in the meta-analysis to synthesize the total UPDRS. Compared to the placebo plus WM group, the severity of PD motor and non-motor symptoms, as evaluated by the total UPDRS score, was significantly improved in the HM plus WM group (n = 308, MD = −8.03, [−10.27, −5.79]; *p* < 0.00001, I^2^ = 0%) (Figure 4A).

*UPDRS Ⅰ*: Three studies (Chen et al., 2014; Ma, 2018; Chen et al., 2020) compared the UPDRS I of HM plus WM group to that of the placebo plus WM. Pooled analysis showed that the HM plus WM group was superior to the placebo plus WM group (n = 240, MD = −0.58 [−0.95, −0.21]; *p* = 0.002, I^2^ = 0%) (Figure 4B).

*UPDRS Ⅱ*: Five trials (Xiao, 2010; Chen et al., 2014; Yu, 2016; Ma, 2018; Chen et al., 2020) studied the effect of the HM plus WM group on the UPDRS II. The meta-analysis revealed that the HM plus WM group was more beneficial for reducing the scores than the placebo plus WM group (n = 196, MD = −2.82, [−4.20, −1.44]; *p* < 0.0001, I^2^ = 0%) (Figure 4C).

**FIGURE 5:**
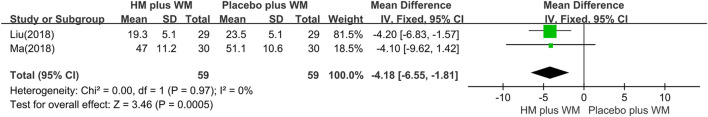
Forest plot of the UPDRS Ⅲ. Subgroup analysis according to types of liver–kidney yin deficiency SD. CI, confidence interval; HM, herbal medicine; MD, mean difference; WM, Western medicine; UPDRS, Unified Parkinson’s Disease Rating Scale; SD, syndrome differentiation.

*UPDRS Ⅲ:* The outcomes of the HM plus WM group versus the placebo plus WM group were reported in eight studies (Jin, 2004a; Xiao, 2010; Chen et al., 2014; Yu, 2016; Yang, 2017; Liu, 2018; Ma, 2018; Chen et al., 2020). The pooled data favored the HM plus WM group (n = 493, MD = −3.85 [−5.07, −2.64]; *p* < 0.00001, I^2^ = 0%) (Figure 4D). Moreover, a subgroup analysis with the same SD was conducted. The results showed that the pooled data also favored the HM plus WM group (n = 118, MD = −4.18 [−6.55, −1.81]; *p* = 0.0005, I^2^ = 0%) (Figure 5).

*UPDRS IV*: Three studies (Xiao, 2010; Chen et al., 2014; Chen et al., 2020) with 204 participants were included in this group. There were no significant differences between the two groups in the UPDRS IV effect size (n = 204, MD = 0.39 [−0.39, 1.18]; *p* = 0.32, I^2^ = 0%) (Figure 4E).

**FIGURE 6:**
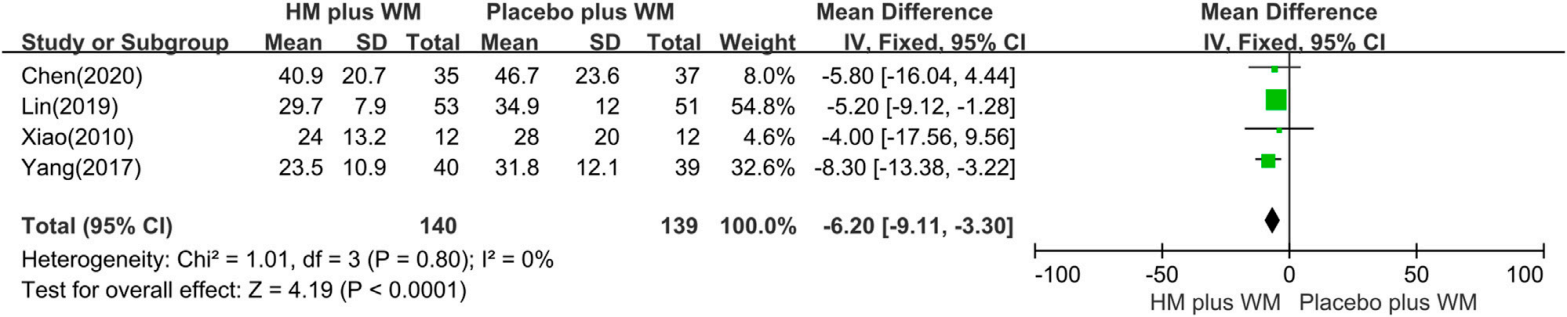
Forest plot of the PDQ-39. CI, confidence interval; HM, herbal medicine; MD, mean difference; WM, Western medicine; PDQ-39, Parkinson’s Disease Questionnaire-39.

*PDQ-39 score*: Four studies (Xiao, 2010; Yang, 2017; Lin et al., 2019; Chen et al., 2020) evaluated the HM plus WM group versus the placebo plus WM group in the treatment of PD. The results showed that the HM plus WM group was better than the placebo plus WM group (n = 279, MD = −6.20 [−9.11, −3.30]; *p* < 0.0001, I^2^ = 0%) (Figure 6).

**FIGURE 7:**
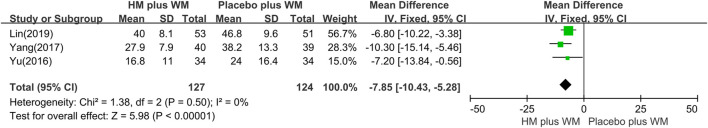
Forest plot of the NMSS. CI, confidence interval; HM, herbal medicine; MD, mean difference; WM, Western medicine; NMSS, Non-Motor Symptoms Scale.

*NMSS*: The pooled data of three studies (Yu, 2016; Yang, 2017; Lin et al., 2019) showed that the HM plus WM group in the NMSS was significantly different between the two groups (n = 251, MD = −7.85 [−10.43, −5.28]; < 0.00001, I^2^ = 0%) (Figure 7).

#### 3.4.2 Adverse events

**FIGURE 8:**
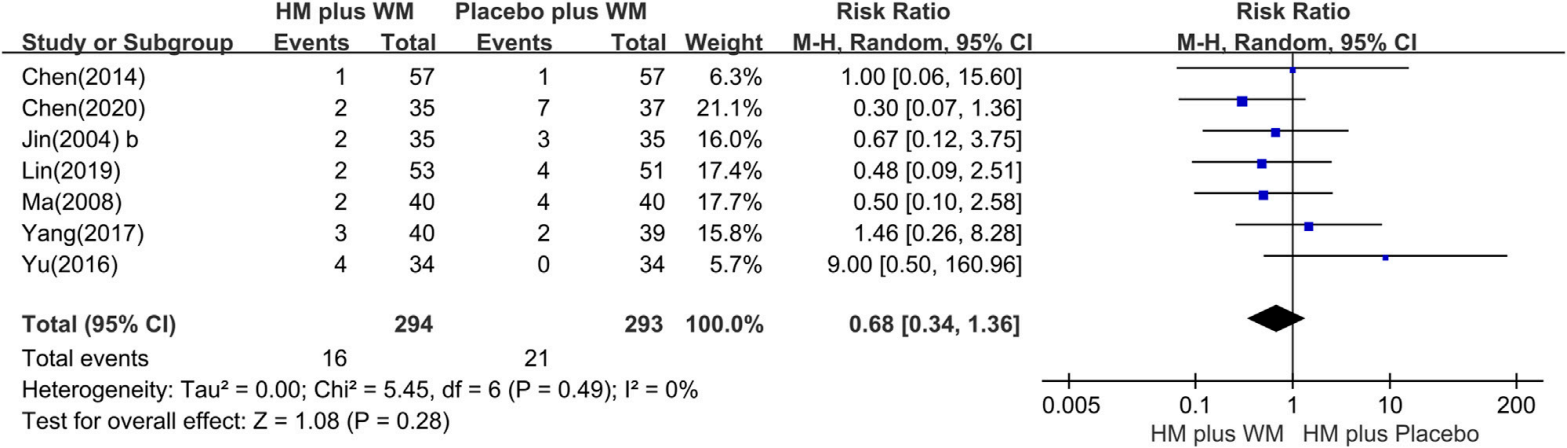
Forest plot of the adverse events. CI, confidence interval; HM, herbal medicine; RR, risk ratio; WM, Western medicine.

Seven RCTs (Jin, 2004a; Jin, 2004b; Ma, 2008; Chen et al., 2014; Yu, 2016; Yang, 2017; Lin et al., 2019; Chen et al., 2020) reported AEs. The meta-analysis showed that there was no statistical difference between the two groups (n = 587, RR = 0.68 [0.34, 1.36], P = 0.28, I^2^ = 0%) (Figure 8). Moreover, the most common adverse reactions of the HM plus WM intervention group were gastrointestinal dysfunction, which included nausea, vomiting, and diarrhea. Dizziness, light dry mouth, and transient increase in creatinine level were also some of its AEs. However, no serious adverse effects were reported in these studies. The most common side effects in the placebo plus WM intervention group were nausea and dizziness. The details of the AEs are shown in Table 3.

**TABLE 3:** Details on adverse events.

First author (years)	Adverse events			
	Intervention group (N)		Control group (N)	
Chen et al. (2014)	Transient increase of creatinine (1)		Hallucination (1)	
Chen et al. (2020)	Dizziness, light dry mouth, and nausea (2)		Light dizziness (2) and nausea (5)	
Jin (2004a)	No AEs		No AEs	
Jin (2004b)	Dizziness (1) and nausea (1)		Abdominal distention (1), lower limb pruritus (1), and xerostomia (1)	
Lin et al. (2019)	Diarrhea (1), nausea, and vomiting (1)		Dizziness (1), gastrointestinal reaction (1), and nausea (2)	
Liu, (2018)	NR		NR	
Ma (2008)	Diarrhea (2)		Anxiety, dyspepsia, insomnia, nausea, and xerostomia (4)	
Ma (2018)	No AEs		No AEs	
Xiao (2010)	NR		NR	
Yang (2017)	Diarrhea (1) and vomiting (2)		Dizziness (1) and vomiting (1)	
Yu (2016)	Nausea and vomiting (4)		No AEs (0)	
Zeng (2015)	NR		NR	

#### 3.4.3 Quality of the evidence

The systematic analysis examined seven outcomes in the intervention and control groups (Supplementary Table S2). Two trials (Jin, 2004a; Jin, 2004b) did not blind the practitioners on the participants and personnel and the outcome assessment section. Moreover, one trial (Zeng, 2015) did not conduct a random sequence generation. We considered the evidence for these outcomes as moderate. For other outcomes, the certainty of the evidence for the outcomes was high.

## 4 Discussion

This is the first SR and meta-analysis that evaluated the efficacy and adverse reactions of HM plus WM treatment based on SD for PD, combined with evidence-quality grading evaluation using the GRADE profiler software. A total of 12 randomized placebo-controlled trials involving 843 patients with PD were selected for meta-analysis, and all studies were conducted in China. The findings of this study were that combination therapy with HM plus conventional WM based on SD for PD showed statistically significant improvement in the assessment of UPDRS scores (total and I–III), PDQ-39, and the NMSS, except for the UPDRS IV score. A significant difference was also observed when compared specifically with the same SD group. In case of the UPDRS IV, there was no statistical difference in the UPDRS IV scores that measure AEs between the two groups. The AEs associated with HM reported in the three studies (Xiao, 2010; Chen et al., 2014; Chen et al., 2020) were dry mouth, internal heat, dry heat, and transient increase in creatinine level. However, these studies commonly mentioned that these symptoms disappeared 1 week later. No serious AEs were reported in the intervention and control groups, indicating that HM was generally safe and well-tolerated in patients with PD. Thus, the present study’s findings support the complementary use of HM based on SD paratherapy in PD.

In previous studies, the most frequent SDs in patients with PD suggested Yin deficiency syndromes in the kidney and liver, qi and blood deficiency, phlegm-heat, wind-stirring, and blood stasis (Hongzhi et al., 2017). In line with this, the frequent SD presented in RCTs included in this review were as follows: “liver–kidney yin deficiency,” from five studies (three single “liver–kidney yin deficiency” (Ma, 2008; Liu, 2018; Ma, 2018), and two combinations with “liver–kidney yin deficiency” (Jin, 2004a; Jin, 2004b)). In addition, the SDs of “stirring wind,” “blood stasis,” “phlegm-heat,” and “Qi blood deficiency” were selected for this SR. To investigate the characteristic subdivided systemic symptoms according to SD, the systemic symptoms of the TEAM questionnaire survey presented in RCTs (Jin, 2004a; Jin, 2004b; Ma, 2008; Yu, 2016) were compared with the characteristics of SD. Moreover, we investigated whether herbal medicines, which were consistent with SD and related systemic symptoms in the TEAM questionnaire survey, were used in each RCT. Moreover, we identified whether HM, which was prescribed based on SD in PD, improved the TEAM systemic symptoms, which is not only limited to PD clinical symptoms. Due to the different names of herbal medicine prescriptions used in each RCT, we checked the herbal ingredients and components of the prescription. The frequent herbal ingredients were *Radix Polygoni Multiflori*, *Gastrodis Tuber*, *Boschniakiae Herba*, and *Rhizoma Ligustici Chuanxiong* (Figure 2). The relationship between the major symptoms in the SD diagnosis of PD and herbal medicine prescriptions used to improve these symptoms was investigated as follows.

The three symptoms, namely, limb spasms (Jin, 2004a; Jin, 2004b; Ma, 2008), stiff neck (Jin, 2004a; Jin, 2004b), and insomnia (Jin, 2004a; Jin, 2004b) in the TEAM questionnaire survey, were improved by HM plus WM in the three RCT studies on patients with PD and diagnosed with a deficiency of the liver and kidney pattern. These three symptoms were usually found in the SD pattern of the liver and kidney deficiency (Hu et al., 2022). The herb ingredient *Radix Polygoni Multiflori* was included in the prescription of the three RCTs and can tonify the liver and kidney, nourish blood and dispel wind, and strengthen the muscles and bones (Chonggang et al., 2002). Moreover, *Radix Polygoni Multiflori* can induce autophagy to prevent human prion protein-mediated neurotoxicity, prevent rotenone-induced apoptosis in SH-SY5Y cells, and enhance α-synuclein-expressing PC12 cell line *in vitro* (Li et al., 2013). Therefore, it is suitable for improving the corresponding SD symptoms of patients with a deficiency of the liver and kidney pattern in PD.

These studies (Jin, 2004a; Jin, 2004b) conducted on back and leg pain in the TEAM questionnaire survey were examined for the effect of HM. Moreover, back and leg pain occurred with stirring wind and blood stasis (Zhu, 2002). The herbal ingredient *Gastrodis Tuber* can be beneficial for qi and blood, dispelling wind, resolving phlegm, and activating the blood and making it clear (Lin et al., 2019). Based on a previous study, *Gastrodis Tuber* inhibits oxidative stress and apoptosis in 1-methyl-4-phenlypyridinum and stimulates SH-SY5Y cells by the upregulation of heme oxygenase through the p38 MAPK/Nrf2 pathway (Chen et al., 2022). Thus, using it to treat SD symptoms, as well as “stirring wind” and “blood stasis,” may be beneficial.

According to three studies (Jin, 2004a; Jin, 2004b; Ma, 2008), night sweats (Jin, 2004a; Jin, 2004b) and back and leg pain (Jin, 2004a; Jin, 2004b; Ma, 2008), which were associated with Yin deficiency (Zhu, 2002), showed that HM plus WM can benefit the systemic symptoms used in SD diagnosis. Thus, *Radix Polygoni Multiflori* can improve the energy metabolism of Yin deficiency syndrome (Gao et al., 2018). Moreover, based on the most frequent herbal components, *Radix Polygoni Multiflori* and *Rhizoma Ligustici Chuanxiong*, five studies (Jin, 2004a; Jin, 2004b; Ma, 2008; Yang, 2017; Lin et al., 2019) reported that *Radix Polygoni Multiflori* is a sovereign herb, while *Rhizoma Ligustici Chuanxiong* is a minister herb. A combination of the the two herbs can improve brain invasion and dredge collaterals (Yang, 2017). In addition, a study (Xu et al., 2014) of tetramethylpyrazine, an alkaloid component extracted from *Rhizoma Ligustici Chuanxiong*, which can activate the blood and dispel stasis and wind, confirmed an improvement in PD symptoms. When MPTP-treated mice were given tetramethylpyrazine bis-nitrone, a derivative of tetramethylpyrazine bis-nitrone, daily for 14 days, the dose dependence reduced the loss of TH-positive nigral cells and lessened striatal dopamine depletion in comparison to the control group.

The TEAM systematic symptoms, SD, and HM presented in RCTs were consistently relevant. However, there is a limitation in that the TEAM questionnaires used in RCTs were different, so further research is needed. Three studies (Jin, 2004a; Jin, 2004b; Ma, 2008) used two different questionnaires in the TEAM symptom assessment, of which two studies (Jin, 2004a; Jin, 2004b) used the same questionnaire named “Self-made Parkinson’s Disease TCM Syndrome Differentiation Scale” (Jin, 2004b), and one study (Ma, 2008) used a different questionnaire named “Diagnosis and curative effect evaluation criteria for senile tremor syndrome in traditional Chinese medicine” (Rong, 1992). Two questionnaires were used to assess five symptoms (limb spasm, stiff neck, insomnia, back and leg pain, and night sweats), using different evaluation questions with different scores.

This SR and meta-analysis had several strengths. We searched both international and Chinese and Korean databases to identify the included articles. Furthermore, the identified studies used common clinical evaluation tools to measure PD. We also used GRADEpro to explore the quality of each outcome. To date, three SRs on the efficacy of HM in PD have been conducted (Wang et al., 2012; Zhang et al., 2015; Shan et al., 2018). To date, few studies have reported HM based on SD, which led to SR’s inability to assess the SD of HM. However, our SR differs from these SRs. In particular, we included new studies and assessed HM based on SD with low heterogeneity.

Despite these positive results, this study has some limitations. First, due to the small number of studies included, there may be publication bias. Moreover, all of the studies were conducted in China, leading to the possibility of publication and regional bias. Further studies are needed to assess other races in other countries. Second, no studies have reported follow-up data. PD is a progressive disease. More outcome measures are needed to assess the long-term effects. Third, due to various SDs, we cannot assess which SD was more effective in treating PD. The existing SD classification has been subdivided into so many different categories. For example, the liver–kidney yin deficiency may include stirring of wind, and since the relationship between the liver and wind is close, the liver is regarded as the wind reservoir in TEAM. Thus, liver–kidney yin deficiency and stirring of wind SD showed symptoms of wind such as tremors due to the liver–kidney yin deficiency, causing increasing hyperactivity of the liver yang (Zhu and Wang, 2011). Therefore, this should be considered in the subgroup analysis of the effectiveness of HM based on the new standardized SD for PD in future studies. Fourth, few studies have reported AEs with specific data, which led to the inability to perform a meta-analysis of AEs. This should be analyzed in future studies. Finally, we could not synthesize the data to assess symptoms due to the use of different questionnaires on the systematic symptoms for SD by the included studies. A questionnaire with accurate reliability and validity should be developed in future studies.

## 5 Conclusion

The efficacy of a combination therapy of HM plus WM based on SD for PD in RCTs was shown and confirmed to reduce AEs than WM plus the placebo. HM treatment may improve motor and non-motor symptoms, as well as quality of life, in patients with PD. It may also be able to compensate for the flaw of standard therapy, which has a limited effect on motor symptoms. Moreover, this study may serve as a basis for SD studies of PD by providing estimates of the treatment effects for individual HM prescriptions and frequently used traditional HM for each SD diagnosis in the future. However, further studies are still needed due to the lack of scientific evidence and existing limitations.

In the future, rigorous research should be performed to improve the methodological quality, and clinical design guidelines must be followed for appropriate evaluation. Further research in China and other countries *via* an international collaboration should be conducted. The development and standardization of SD diagnostic methods targeting PD have not been established. Thus, research on SD according to patient symptoms should be conducted to contribute to developing PD diagnoses and treatment in traditional medicine.

## Data Availability

The original contributions presented in the study are included in the article/Supplementary Material; further inquiries can be directed to the corresponding authors.
