# Supplementary material for: Efficacy of herbal medicine treatment based on syndrome differentiation for Parkinson’s disease: A systematic review and meta-analysis of randomized placebo-controlled clinical trials
Source: Front Pharmacol. 2023 Feb 27;14:1108407. doi: 10.3389/fphar.2023.1108407 (PMC10012343; doi:10.3389/fphar.2023.1108407)
Supplement: Supplementary file 1 [file DataSheet1.docx]

Supplementary Material

Efficacy of Herbal Medicine Treatment Based on Syndrome Differentiation for Parkinson’s Disease: A Systematic Review and Meta-analysis of Randomized Placebo-controlled Clinical Trials

Purumea Jun^1†^, HuiYan Zhao^1,2†^, In Chul Jung^3^, Ojin Kwon^1^, Chang-Hyun Han^1,*^, Jiyoon Won ^1,*^, Jung-Hee Jang^1,*^

*** Correspondence:**Jung-Hee Jang
[jee3838@kiom.re.kr](mailto:jee3838@kiom.re.kr)

Jiyoon Won
[erigh15@kiom.re.kr](mailto:erigh15@kiom.re.kr)

Chang-Hyun Han
[chhan@kiom.re.kr](mailto:chhan@kiom.re.kr)

# Supplementary Table 1 Referred Reporting Items for Systematic Review and Meta-analysis 2020 Checklist

| **Section and Topic** | **Item #** | **Checklist item** | **Location where item is reported** |
| --- | --- | --- | --- |
| **TITLE** | | |  |
| Title | 1 | Identify the report as a systematic review. | Page 1 |
| **ABSTRACT** | | |  |
| Abstract | 2 | See the PRISMA 2020 for Abstracts checklist. | All YES |
| **INTRODUCTION** | | |  |
| Rationale | 3 | Describe the rationale for the review in the context of existing knowledge. | Page 2 |
| Objectives | 4 | Provide an explicit statement of the objective(s) or question(s) the review addresses. | Page 2-3 |
| **METHODS** | | |  |
| Eligibility criteria | 5 | Specify the inclusion and exclusion criteria for the review and how studies were grouped for the syntheses. | Page 4-5 |
| Information sources | 6 | Specify all databases, registers, websites, organisations, reference lists and other sources searched or consulted to identify studies. Specify the date when each source was last searched or consulted. | Page 4-5 |
| Search strategy | 7 | Present the full search strategies for all databases, registers and websites, including any filters and limits used. | Page 3 |
| Selection process | 8 | Specify the methods used to decide whether a study met the inclusion criteria of the review, including how many reviewers screened each record and each report retrieved, whether they worked independently, and if applicable, details of automation tools used in the process. | Page 4 |
| Data collection process | 9 | Specify the methods used to collect data from reports, including how many reviewers collected data from each report, whether they worked independently, any processes for obtaining or confirming data from study investigators, and if applicable, details of automation tools used in the process. | Page 4 |
| Data items | 10a | List and define all outcomes for which data were sought. Specify whether all results that were compatible with each outcome domain in each study were sought (e.g. for all measures, time points, analyses), and if not, the methods used to decide which results to collect. | Page 4 |
|  | 10b | List and define all other variables for which data were sought (e.g. participant and intervention characteristics, funding sources). Describe any assumptions made about any missing or unclear information. | Page 5 |
| Study risk of bias assessment | 11 | Specify the methods used to assess risk of bias in the included studies, including details of the tool(s) used, how many reviewers assessed each study and whether they worked independently, and if applicable, details of automation tools used in the process. | Page 4 |
| Effect measures | 12 | Specify for each outcome the effect measure(s) (e.g. risk ratio, mean difference) used in the synthesis or presentation of results. | Page 4 |
| Synthesis methods | 13a | Describe the processes used to decide which studies were eligible for each synthesis (e.g. tabulating the study intervention characteristics and comparing against the planned groups for each synthesis (item #5)). | Page 4-5 |
|  | 13b | Describe any methods required to prepare the data for presentation or synthesis, such as handling of missing summary statistics, or data conversions. | Page 5 |
|  | 13c | Describe any methods used to tabulate or visually display results of individual studies and syntheses. | Page 4-5 |
|  | 13d | Describe any methods used to synthesize results and provide a rationale for the choice(s). If meta-analysis was performed, describe the model(s), method(s) to identify the presence and extent of statistical heterogeneity, and software package(s) used. | Page 4 |
|  | 13e | Describe any methods used to explore possible causes of heterogeneity among study results (e.g. subgroup analysis, meta-regression). | Page 4 |
|  | 13f | Describe any sensitivity analyses conducted to assess robustness of the synthesized results. | Page 4 |
| Reporting bias assessment | 14 | Describe any methods used to assess risk of bias due to missing results in a synthesis (arising from reporting biases). | Page 4 |
| Certainty assessment | 15 | Describe any methods used to assess certainty (or confidence) in the body of evidence for an outcome. | Page 5 |
| **RESULTS** | | |  |
| Study selection | 16a | Describe the results of the search and selection process, from the number of records identified in the search to the number of studies included in the review, ideally using a flow diagram. | Page 5 |
|  | 16b | Cite studies that might appear to meet the inclusion criteria, but which were excluded, and explain why they were excluded. | Page 5-6 |
| Study characteristics | 17 | Cite each included study and present its characteristics. | Table 1 |
| Risk of bias in studies | 18 | Present assessments of risk of bias for each included study. | Figure 3 |
| Results of individual studies | 19 | For all outcomes, present, for each study: (a) summary statistics for each group (where appropriate) and (b) an effect estimate and its precision (e.g. confidence/credible interval), ideally using structured tables or plots. | Table 1 |
| Results of syntheses | 20a | For each synthesis, briefly summarise the characteristics and risk of bias among contributing studies. | Page 6 |
|  | 20b | Present results of all statistical syntheses conducted. If meta-analysis was done, present for each the summary estimate and its precision (e.g. confidence/credible interval) and measures of statistical heterogeneity. If comparing groups, describe the direction of the effect. | Page 6 |
|  | 20c | Present results of all investigations of possible causes of heterogeneity among study results. | Page 6 |
|  | 20d | Present results of all sensitivity analyses conducted to assess the robustness of the synthesized results. | Page 6 |
| Reporting biases | 21 | Present assessments of risk of bias due to missing results (arising from reporting biases) for each synthesis assessed. | Page 6 |
| Certainty of evidence | 22 | Present assessments of certainty (or confidence) in the body of evidence for each outcome assessed. | Page 7 |
| **DISCUSSION** | | |  |
| Discussion | 23a | Provide a general interpretation of the results in the context of other evidence. | Page 7 |
|  | 23b | Discuss any limitations of the evidence included in the review. | Page 8-9 |
|  | 23c | Discuss any limitations of the review processes used. | Page 8-9 |
|  | 23d | Discuss implications of the results for practice, policy, and future research. | Page 9 |
| **OTHER INFORMATION** | | |  |
| Registration and protocol | 24a | Provide registration information for the review, including register name and registration number, or state that the review was not registered. | Page 3 |
|  | 24b | Indicate where the review protocol can be accessed, or state that a protocol was not prepared. | Page 3 |
|  | 24c | Describe and explain any amendments to information provided at registration or in the protocol. | Page 3 |
| Support | 25 | Describe sources of financial or non-financial support for the review, and the role of the funders or sponsors in the review. | Page 10 |
| Competing interests | 26 | Declare any competing interests of review authors. | Page 9 |
| Availability of data, code and other materials | 27 | Report which of the following are publicly available and where they can be found: template data collection forms; data extracted from included studies; data used for all analyses; analytic code; any other materials used in the review. | None |

# Supplementary Table 2 Risk of bias assessment

| Category | Content |
| --- | --- |
| Selection bias | Low risk of bias: the study authors performed sequence generation by using computer random number generation or random number tables. |
|  | High risk of bias: the study authors performed that sequence generation by using visiting sequence. |
|  | Unclear risk of bias: the study did not mention the sequence method. |
| Performance bias | Low risk of bias: the study authors performed the blinding of participants and personnel. |
|  | High risk of bias: The authors performed the blinding of participants or personnel alone. |
|  | Unclear risk of bias: The authors did not mention blinding methods. |
| Detection bias | Low risk of bias: blinding of outcome assessment ensured, and blinding could not be broken. |
|  | High risk of bias: no blinding of outcome assessment, or the outcome measurement can be able to influence by lack of blinding. |
|  | Unclear risk of bias: The authors did not mention about outcome assessment method. |
| Attrition bias | Low risk of bias: There were no missing data, or the missing data were unlikely to have treatment effects. However, the study method was sufficient. |
|  | High risk of bias: There was missing data, which made attrition bias. |
|  | Unclear risk of bias: There were insufficient methods to assess the missing data. |
| Reporting bias | Low risk of bias: The trials had serious adverse events, and there was an original trial protocol, which has been registered on the website. |
|  | High risk of bias: There reported one or more predefined outcomes. |
|  | Unclear risk of bias: Not all predefined outcomes were reported fully, nor was it reported whether the protocol was registered. |
| Other bias | Low risk of bias: The studies reported a calculated sample size. |
|  | High risk of bias: The studies reported the wrong sample size. |
|  | Unclear risk of bias: The studies did not report on the sample size method. |

# Supplementary Table 3 The Grading of Recommendations Assessment, Development, and Evaluation assessment

| High certainty | We are very confidence that true effect lies close to that of the estimate of the effect |
| --- | --- |
| Moderate certainty | We are moderate confidence in the effect estimate: the true effect is likely to be close the estimate of the effect, but there is possibility that it is substantially different. |
| Low certainty | The effect estimate is limited, the true effect may be substantially different from the estimate of the effect |
| Very low certainty | We have very little confidence in the effect estimate: the true effect is likely to be substantially different from the estimate of effect |

# Supplementary Table 4 Quality of evidence assessment for HM plus WM versus HM plus placebo

| Certainty assessment | | | | | | | No. of participants | | Effect | | Certainty | Importance |
| --- | --- | --- | --- | --- | --- | --- | --- | --- | --- | --- | --- | --- |
| No of studies | Study  design | Risk of bias | Inconsistency | Indirectness | Imprecision | Other consideration | ACE | EA | Relative  (95% CI) | Absolute  (95% CI) |  |  |
| ***Total UPDRS*** | | | | | | | | | | | | |
| 5 | Randomized trials | Serious^a^ | Not serious | Not serious | Not serious | None | 257 | 251 | – | MD **5.31 lower** (8.17 lower to 2.46 lower) | ⨁⨁⨁◯ Moderate | CRITICAL |
| ***UPDRS Ⅰ*** | | | | | | | | | | | | |
| 3 | Randomized trials | Not serious | Not serious | Not serious | Not serious | None | 122 | 118 | – | MD **0.58 lower** (0.95 lower to 0.21 lower) | ⨁⨁⨁⨁ High | CRITICAL |
| ***UPDRS Ⅱ*** | | | | | | | | | | | | |
| 5 | Randomized trials | Not serious | Not serious | Not serious | Not serious | None | 146 | 150 | – | MD **2.82 lower** (4.2 lower to 1.44 lower) | ⨁⨁⨁⨁ High | CRITICAL |
| ***UPDRS Ⅲ*** | | | | | | | | | | | | |
| 9 | Randomized trials | Serious^b^ | Not serious | Not serious | Not serious | None | 280 | 283 | – | MD **3.6 lower** (4.79 lower to 2.4 lower) | ⨁⨁⨁◯ Moderate | CRITICAL |
| ***UPDRS Ⅳ*** | | | | | | | | | | | | |
| 3 | Randomized trials | Not serious | Not serious | Not serious | Not serious | None | 104 | 100 | – | MD **0.39 higher** (0.39 lower to 1.18 higher) | ⨁⨁⨁⨁ High | CRITICAL |
| ***PDQ-39*** | | | | | | | | | | | | |
| 4 | Randomized trials | Not serious | Not serious | Not serious | Not serious | None | 140 | 139 | – | MD **6.2 lower** (9.11 lower to 3.3 lower) | ⨁⨁⨁⨁ High | CRITICAL |
| ***NMSS*** | | | | | | | | | | | | |
| 3 | Randomized trials | Not serious | Not serious | Not serious | Not serious | None | 127 | 124 | – | MD **7.85 lower** (10.43 lower to 5.28 lower) | ⨁⨁⨁⨁ High | CRITICAL |
| Adverse event | | | | | | | | | | | | |
| 7 | Randomized trials | Serious^b^ | not serious | not serious | not serious | none | 16/294 (5.4%) | 21/293 (7.2%) | RR 0.68 | **23 fewer per 1,000** (from 47 fewer to 26 more) | ⨁⨁⨁◯ Moderate | IMPORTANT |

a: One study failed for “Random sequence generation”; b: Two studies failed for “Blinding of participant and personnel data” and “Blinding of outcome assessment”; HM: herbal medicine; NMSS: Non-Motor Symptom Scale; UPDRS: Unified Parkinson’s Disease Rating Scale score; PDQ-39: Parkinson’s Disease Questionnaire-39; WM: Western medicine
